# Computational Approaches to Penicillin Allergy De-labelling: A Clinical Review

**DOI:** 10.1007/s12016-026-09203-0

**Published:** 2026-09-22

**Authors:** Shuayb Elkhalifa, Jack Devey, Abdulrahaman Al-Asali, Haggar Elbashir, Maja Bulatović Ćalasan, Rick G. Pleijhuis, Ingrid Terreehorst, Fulvio Salvo, Irfan Shafiq, Said Isse, Mohamed Abuzakouk, Mohamed Medhat Gaber, Rehan Bhana

**Affiliations:** 1 Cleveland Clinic Abu Dhabi, Abu Dhabi, United Arab Emirates; 2 University of Manchester, Manchester, UK; 3 Birmingham City University, Birmingham, UK; 4 Lancashire Teaching Hospitals NHS Foundation Trust, Preston, UK; 5 University Medical Center Utrecht, Utrecht, Netherlands; 6 Diakonessenhuis Utrecht, Amsterdam University Medical Center, Zaans Medisch Centrum, Amsterdam, Netherlands; 7 Cairns Hospital, Cairns and Hinterland Hospital and Health Service, Cairns, QLD Australia; 8 James Cook University, Townsville, QLD Australia; 9 Queensland University of Technology, Brisbane, Queensland Australia

**Keywords:** Penicillin allergy, Drug allergy, De-labelling, Machine learning, Natural language processing, Clinical decision support, Antimicrobial stewardship

## Abstract

Penicillin allergy is recorded in roughly one in ten hospital patients, yet fewer than one in ten of those records reflects a real, clinically significant allergy. The mismatch matters: patients with a penicillin label receive more second-line and broader-spectrum antibiotics, with measurable downstream costs in infection, surgical site morbidity, length of stay, and antimicrobial resistance. Specialist-led skin testing and graded oral challenge remain the diagnostic standard, but the global allergy workforce cannot test the population that needs testing. Validated decision rules such as PEN-FAST, structured non-allergist pathways, and direct oral challenge in low-risk patients have closed part of that gap, but uptake is still limited by inconsistent free-text documentation, variable clinician confidence, and weak integration with electronic health records. This review summarises how computational methods are being added to that pathway. Supervised machine-learning models can mine electronic health records to identify candidates for review. Natural language processing can recover the timing, character, and severity of a reaction from clinic letters that the structured allergy field omitted. Predictive models that combine clinical, laboratory, and immunological inputs are starting to outperform rule-based scores on beta-lactam risk. Decision-support systems are moving past simple drug-name matching toward context-aware alerts. Image classifiers and large language models are opening a route to rapid, photograph-and-history triage. We discuss what is needed for any of this to be safe in routine care: representative training data, transparent model behaviour, regulatory alignment, and prospective evaluation against the outcomes the antimicrobial stewardship community already measures.

## Key Points

Most patients labelled as penicillin-allergic are not, and the resulting use of broader-spectrum antibiotics drives infections, costs, and resistance. Computational tools, ranging from rule-based mobile applications to language models, predictive risk scores, and image classifiers, are now plausible adjuncts that can extend safe de-labelling beyond the small global allergist workforce, provided they are deployed with explicit clinician oversight and prospective evaluation.

## Introduction

Penicillin allergy de-labelling is the structured removal of an inaccurate or outdated penicillin allergy entry from a patient’s record after history-based risk assessment and, where appropriate, drug challenge. Around 10% of inpatients carry such a label, but fewer than 1% are confirmed allergic on formal evaluation [1]. The clinical and operational consequences of that error are well described. Patients with a penicillin label are more likely to receive vancomycin, fluoroquinolones, clindamycin, and other agents associated with greater toxicity, more Clostridioides difficile infection, longer admissions, more surgical-site infection, and higher costs [2, 3].

National guidance, including NICE CG183 in the United Kingdom and the BSACI consensus on non-allergist de-labelling, now treats penicillin allergy as a stewardship problem and not solely an allergy problem [4, 5]. In the United States, the Infectious Diseases Society of America and the Society for Healthcare Epidemiology of America likewise place penicillin allergy evaluation within antimicrobial stewardship, and allergy assessment is a recognised activity of the Infectious Diseases Society of America Antimicrobial Stewardship Centers of Excellence [6]. Validated rules such as PEN-FAST identify low-risk patients who can proceed to direct oral challenge without prior skin testing [7]. PEN-FAST was derived and validated in adults, and emerging paediatric data suggest that it can also support assessment in children [8]. Structured pathways delivered by pharmacists, anaesthetists, infectious diseases teams, and general physicians have shown that de-labelling outside the allergy clinic is safe at scale [9–11]. The remaining bottlenecks are not clinical. They are documentation quality, workflow fit, clinician confidence, and the limited number of allergists [10, 12].

Computational methods are starting to address those bottlenecks. Supervised models (programs trained on labelled examples so they can flag similar cases in new data) can flag candidates for review across whole inpatient populations [13]. Natural language processing (NLP), software that interprets free text, can read free-text clinical notes that the coded allergy field cannot [14–16]. Predictive scores can stratify risk using inputs that simple rules do not consider [17]. Decision-support systems can suppress nuisance allergy alerts and surface relevant ones [18, 19]. Image-based models, and large language models that combine image and text, could enable rapid triage without a face-to-face specialist appointment.

This article reviews the computational methods that have been published or piloted for penicillin allergy de-labelling, summarises the evidence behind them, and sets out what clinicians and developers need to do for these tools to move from prototype to routine practice.

## The Clinical Problem and the Gaps in the Existing Pathway

Most penicillin allergy entries are inherited rather than recently confirmed. They originate from non-specific events in childhood, a rash, gastrointestinal upset, or family report, that were never formally evaluated and are then carried in the electronic health record (EHR) for decades [1, 12]. The structured allergy field is rarely informative. Free-text descriptions, when present, often record only ‘rash’, ‘reaction’, or ‘unknown’, with no timing or severity. That single entry can shape the antibiotics a patient is offered for many years.

The traditional allergy work-up, which combines a detailed history, skin prick and intradermal testing, and a supervised oral challenge, remains the diagnostic standard, but it cannot be delivered at the scale that the mislabelled population requires. Even high-resource health systems do not have enough allergists to test the mislabelled population, and most countries have far fewer. PEN-FAST and similar rules were designed precisely to identify the large subset of patients who can be assessed and challenged by non-specialists in a few minutes [7]. Implementation studies in the United States, United Kingdom, Australia, and Canada confirm that this works when the workflow is in place [9–11, 20].

Three structural barriers persist. First, allergy documentation is too thin to feed any decision tool, whether human or computational, without manual cleaning. Second, even when a tool is validated, embedding it into the order-entry path of the EHR is non-trivial and varies by vendor and institution. Third, allergists, pharmacists, and prescribers vary in their willingness to act on a low-risk classification without face-to-face assessment. Computational methods can help with the first two and can support, but not replace, the third.

The economic case is by now well-quantified. Patients with a recorded penicillin allergy in US health systems incur higher antibiotic costs, longer admissions, and an excess rate of C. difficile, surgical site infection, and methicillin-resistant Staphylococcus aureus acquisition compared to label-matched controls without an allergy record [2, 3, 21]. Most of those harms are mediated by avoidable second-line antibiotic exposure rather than by the allergy itself. Any tool that increases the volume of accurate de-labelling therefore acts directly on antimicrobial stewardship endpoints that hospitals already report.

The point that has the strongest implications for any computational claim in this space is the asymmetry of error. False positives, labelling a non-allergic patient as allergic, are common, ongoing, and damaging at population level, but they are invisible. False negatives, declaring a truly allergic patient as low-risk and giving them penicillin, are rare but acutely visible. A model trained on data that already over-classifies allergy will be safe but of little practical value. A model trained to maximise de-labelling without explicit risk thresholds will be useful but unsafe. Every method discussed below has to be evaluated against that asymmetry.

## Computational Methods Applied to Penicillin De-labelling

Box 1. Glossary of key terms used in this review

**Table:** 

Term	Plain-language meaning
Supervised machine learning	A computer program that learns from many labelled examples so it can recognise similar patterns in new patient records.
Natural language processing (NLP)	Software that reads free text, such as clinic letters, and pulls out specific facts like the type and timing of a reaction.
Predictive risk model	A calculation that combines several patient details to estimate the chance of a true allergy, giving a more individual result than a fixed rule.
Clinical decision support	Prompts or alerts built into the prescribing system that give the clinician relevant information at the moment of prescribing.
Convolutional neural network (CNN)	A type of image-recognition model that can classify photographs, for example of a skin reaction.
Large language model (LLM)	A system trained on very large amounts of text that can summarise records and answer questions in plain language.
Federated learning	A way of training a model across several hospitals without moving patient data out of each hospital.
Discrimination and calibration	Two measures of model performance. Discrimination, often reported as the area under the curve, is how well a model separates allergic from non-allergic patients. Calibration is how closely predicted risks match observed outcomes.
Hallucination	When a language model produces fluent text that sounds correct but is factually wrong.
Retrieval grounding	Linking a model output back to the source record so that statements can be checked against the original data.

Computational tools now sit across the de-labelling pathway: case-finding, history reconstruction, risk stratification, decision support at the point of prescribing, and image-based triage of cutaneous reactions (Table 1).

**Table 1:** Computational methods applied to penicillin allergy de-labelling

Method	Typical input	Where it sits in the pathway	Representative evidence	Main limitation	Plain-language example
Rule-based decision tools (e.g. PEN-FAST, DrugAllergy 1.0)	Structured history items	Triage / non-specialist assessment	Trubiano [7]; Elkhalifa [23]; Arora [48]	Cannot adapt to new evidence without re-coding	A nurse answers a set of fixed questions in a smartphone app; the app scores the answers and shows whether the patient is low risk and can be offered a direct oral challenge.
Supervised machine learning for case-finding	Coded EHR fields plus laboratory values	Population-level identification of candidates for review	Jiang [13]; Inglis [14]; Stanekova [15]; Molloy [49]	External validation and class imbalance	Software scans every inpatient record overnight and produces a daily list of patients with a penicillin label who look suitable for review.
Natural language processing for history extraction	Free-text clinic letters and notes	Recovering reaction detail from unstructured records	Khurana [16]; Suh [26]; Botero-Aguirre [50]	Institution-specific vocabulary; handling of negation	Software reads a clinic letter and identifies that the reaction was a mild rash at age five, information that was missing from the coded allergy field.
Predictive risk models	Structured, laboratory and immunological data	Refined risk stratification beyond fixed rules	Núñez [17]; Schuurmans [27]	Small, single-centre training datasets	A model combines age, previous antibiotic tolerance and blood results to give a more individual estimate of true allergy than a fixed rule.
Clinical decision support / alert refinement	Allergy entry plus the drug being prescribed plus patient context	Point-of-prescribing safety net	Graafsma [18]; De Vito [19]	Risk of alert fatigue if context is incomplete	When a doctor prescribes amoxicillin, the system checks the record, sees that the patient later tolerated amoxicillin, and suppresses an unnecessary allergy warning.
Image-based and multi-modal models	Photograph of the reaction plus EHR text	Triage of cutaneous reactions; asynchronous review	Lee [34]; Wiest [35]	Image quality; need for human review of language-model output	A patient photographs a rash before the visit; software offers a provisional classification that the clinician then reviews.

### Rule-based Decision Tools

The first wave of digital support for non-allergist de-labelling was rule-based. Clinical decision rules such as PEN-FAST were re-implemented as web and smartphone tools, often with embedded prompts to standardise the history [7]. PEN-FAST itself was derived and validated in over 600 adult patients and identified a low-risk group with a positive challenge rate below 1%, which is the operating point that has anchored most subsequent non-allergist pathways. External validation in an independent Asian cohort has since confirmed the rule’s high negative predictive value but only modest overall discrimination (area under the curve 0.66), reinforcing that a rule derived in one population may not transfer unchanged to another [22]. The Drug Allergy mobile application developed by several of the authors is one example of an expert-system implementation: a clinician-designed questionnaire encoded as an explicit decision tree that classifies the patient as low, moderate, or high risk and links to recommended next steps [23]. Rule-based systems are inflexible by design, they only do what their authors programmed, but their behaviour is fully transparent, easy to audit, and easy to align with national guidance. They remain the most widely deployed digital tool in this area and are the reasonable benchmark against which newer methods should be judged. Any model that does not at least match a current rule on safety while improving throughput, equity, or documentation quality is unlikely to be worth deploying.

### Supervised Machine Learning for Case-finding

Supervised models trained on EHR data can identify inpatients with penicillin labels who are likely candidates for review. Jiang et al. reported a deep-learning case-finder that flagged low-risk inpatients in real time within a routine hospital workflow, allowing prospective de-labelling rather than retrospective audit [13]. Inglis et al. trained a supervised NLP classifier to categorise penicillin reactions and stratify risk directly from free-text records [14]; Stanekova et al. extended this with synthetic data and transfer learning to compensate for the small size of curated allergy datasets [15]. A systematic review of supervised models for adverse drug reactions across hospital populations concluded that, on structured inputs, these models often beat rule-based predictors on discrimination, although calibration and external validation were uneven [24].

Two methodological points are particularly relevant for de-labelling. First, the prevalence of true allergy in any unselected population is low, so naive accuracy is misleading; sensitivity for severe reactions and the negative predictive value among patients flagged as low-risk are the operational metrics that should be reported. Second, most published models have been trained and tested at the same institution, on the same documentation conventions, and with the same case mix. Performance on transfer to a different EHR vendor or a different country has, where reported, been markedly lower [24, 25]. Multi-centre training and prospective external validation are still rare in this literature.

### Natural Language Processing for History Extraction

The structured allergy field is unreliable; the free text is where the information lives. NLP techniques recover timing, severity, exposure, and prior tolerance from clinic letters, discharge summaries, and pre-anaesthetic assessments. Khurana et al. summarised the state of clinical NLP and the recurring difficulties of linguistic variation, abbreviation, and negation [16]. In a directly relevant application, Suh et al. showed that an NLP engine could extract pre-anaesthetic history elements, including allergy-related information, from narrative records with high accuracy [26]. The clinical utility of NLP in this space is not in replacing clinicians; it is in restoring information that has already been written down but is not retrievable from the structured record.

### Predictive Risk Models

Static decision rules treat all low-risk patients identically. Predictive models can refine that estimate by incorporating age, prior antibiotic tolerance, comorbidity, and laboratory values. Núñez et al. reported a neural-network model for true beta-lactam allergy that achieved 89.5% sensitivity and 86.1% specificity, outperforming standard statistical methods on the same dataset [17]. Comparable approaches are being explored for non-steroidal anti-inflammatory drug hypersensitivity, perioperative reactions, and severe delayed reactions. Cardiac anaesthesia studies of data-driven early warning during surgery, including the HYPE-2 trial, illustrate the implementation pattern such tools will need to follow before they are accepted at the point of care [27, 28].

### Clinical Decision Support and Alert Refinement

Allergy alerts in EHR-based prescribing systems are notorious for false positives and alert fatigue. Studies of inpatient prescribing repeatedly find override rates above 90% for medication-allergy alerts, with most of the overridden alerts judged appropriate by the prescriber. Recent work has tried to make them less noisy and more useful. A scoping review by Graafsma et al. mapped how machine-learning techniques can refine medication alerts using patient-specific factors, including prior tolerance, the specific drug name, and the documented severity, reducing irrelevant interruptions while preserving safety-critical warnings [18]. The HELIOT prototype combined free-text reaction history with structured pharmaceutical data, including prior tolerance and excipient composition, and reported improved precision and recall over conventional rule-based alerting on a synthetic patient set [19]. A general review of decision-support systems concluded that the gains are real, but contingent on clean data and clinician trust [29]. The point at which alert refinement becomes a de-labelling tool is when the system is allowed to write back to the allergy field, for example, by suggesting that a recorded penicillin allergy be downgraded after documented uneventful tolerance of amoxicillin during a previous admission. Whether and how that write-back is permitted is now as much a governance question as a technical one.

### Image-based Models for Cutaneous Reactions

Cutaneous findings often anchor the original allergy label and are interpreted variably between clinicians. Convolutional neural networks (CNNs), since the U-Net and post-AlexNet generation of architectures, have produced strong results in medical image segmentation and classification [30–33]. In allergy specifically, attention-U-Net models segmented skin-prick wheal and erythema from smartphone images with accuracy of 99.8% and 96.6% respectively [34]. The clinical attraction is that an image of a current or photographed historical reaction can be classified asynchronously, before the patient is seen, and the classification can support the history-taking that follows. The risks are familiar from dermatology: training sets that under-represent darker skin tones produce models that perform less well in those patients, and image quality from a patient’s own phone is variable and difficult to standardise. Reporting against the relevant skin-tone diversity benchmarks is now expected of any image-based dermatology model.

### Large Language Models and Multi-modal Triage

**Fig. 1:**
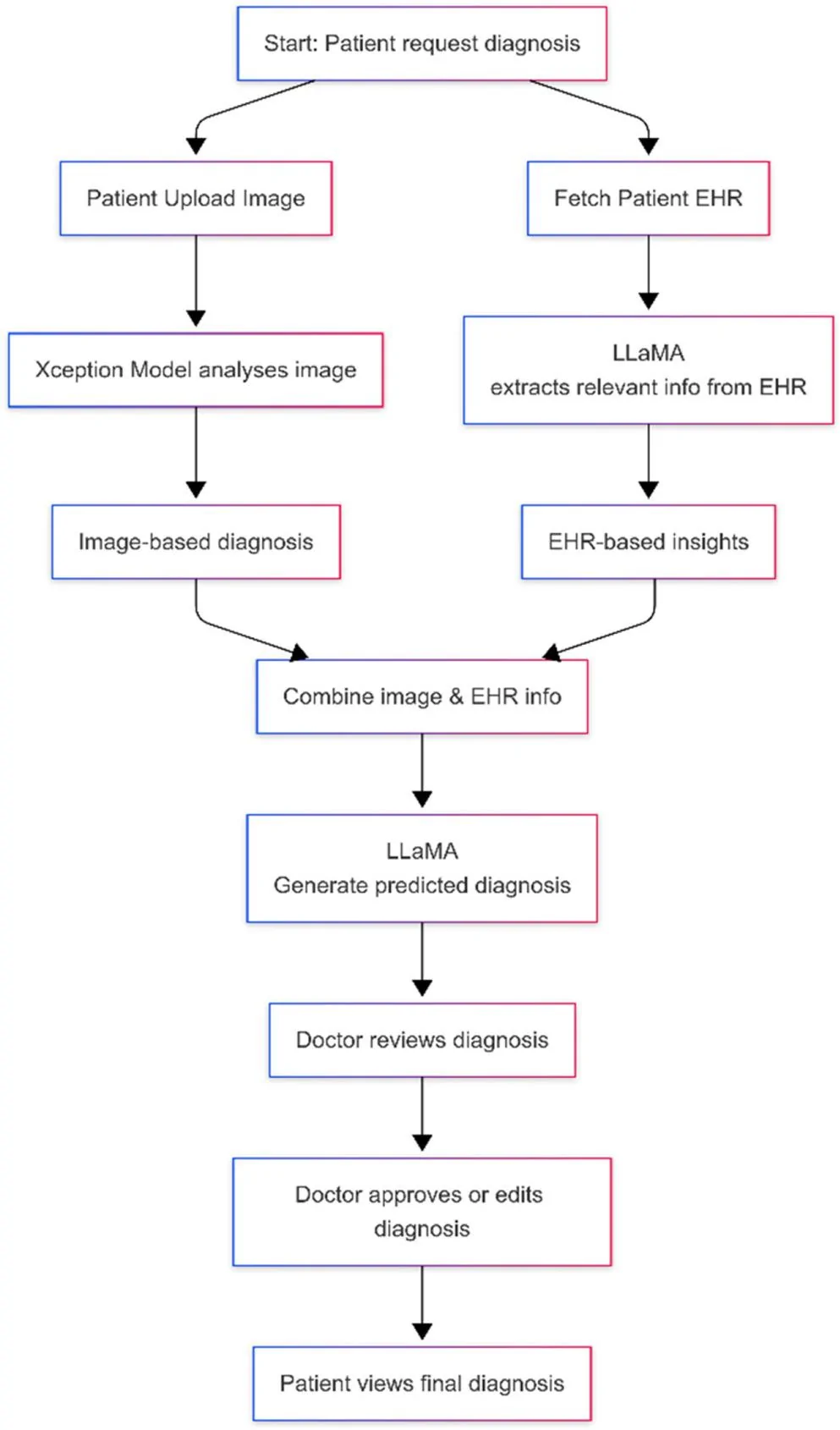
Proposed multi-modal computational diagnostic workflow for penicillin allergy assessment. The patient uploads a photograph of a reported reaction and authorises access to the electronic health record. A convolutional neural network classifies the image; a large language model summarises the relevant history. The two outputs are fused and presented to the responsible clinician, who confirms or overrides the recommendation before any change to the allergy record

**Fig. 2:**
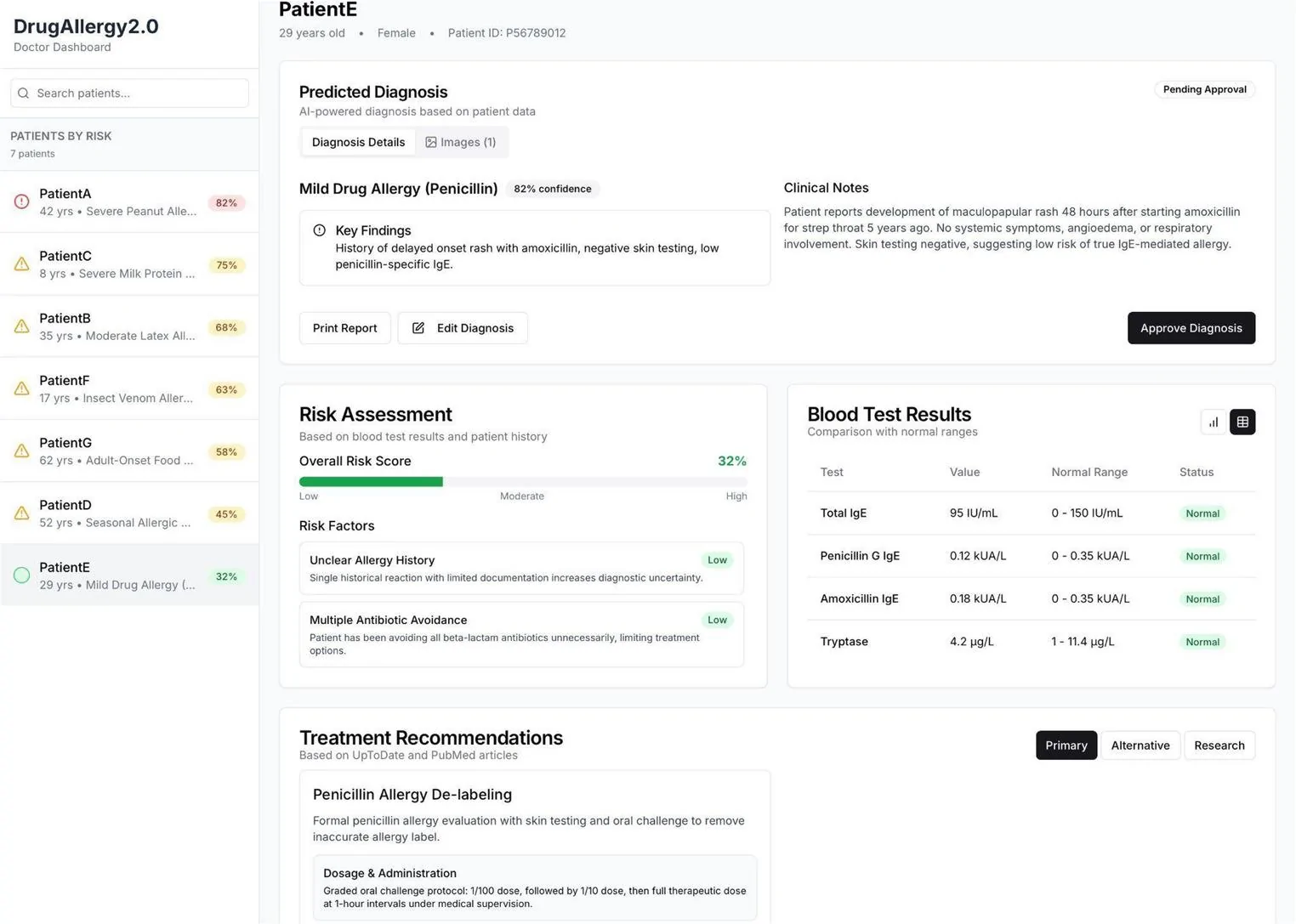
Clinician- and patient-facing prototypes for the next-generation Drug Allergy application. The clinician dashboard prioritises patients by risk score, displays the contributing features, and links to evidence-based recommendations. The patient-facing view supports image upload, asynchronous review, and structured follow-up. Shown as a design concept; the system has not been prospectively validated

Large language models (LLMs) are the most recent addition to this toolkit. Evaluations of clinical LLMs report at least 90% accuracy in identifying explicit and implicit clinical findings from notes in zero-shot prompting, and over 98% accuracy when extracting data from synthetic EHRs containing both structured and unstructured fields [35, 36]. The plausible near-term use is multi-modal: a photograph of a reported drug reaction is classified by a convolutional neural network, a language model summarises the patient’s record, the two outputs are presented together to a clinician who confirms or overrides the suggestion (Fig. 1). Several of the authors have used this scaffold to design a clinician- and patient-facing prototype for the next iteration of the Drug Allergy application (Fig. 2), which is presented as a worked example of the workflow rather than a validated product. The known failure mode of LLMs in this setting is hallucination, the production of fluent, plausible, but fabricated content, particularly when the underlying record is sparse. Any LLM-derived summary that is allowed to alter an allergy entry must therefore be paired with retrieval grounding to the source record and with an explicit clinician sign-off step.

## Implementation, Governance, and Equity

The barriers to adoption are no longer mainly technical. They are sociotechnical. A recent narrative review of computational methods across allergy and immunology reached the same conclusion, observing that although publications in this field are rising, no computationally enabled medical device had yet been approved specifically for allergy and immunology, and proposing a six-point road map, from prioritising high-impact applications and defining clinically relevant benchmarks to rigorous governance, operationalisation, trustworthy adoption, and life-cycle management, to close the research-to-implementation gap [37].

### Bias and Representation

Most published allergy datasets come from a small number of academic centres in North America, Europe, and Australasia. Models trained on these data may not transfer to populations they were not trained on, and may understate or overstate risk in ethnic minorities and other under-represented groups [38, 39]. The minimum standard now expected is reporting against CONSORT-AI and SPIRIT-AI, with explicit description of training and validation populations and disaggregated performance [40].

### Explainability

Clinicians do not have to understand every layer of a neural network, but they do need to know which patient features drove a given recommendation. Attention-based and BERT-style architectures, SHAP values, and counterfactual explanations all reduce the black-box problem to varying degrees [40, 41]. For penicillin de-labelling, where a wrong call risks anaphylaxis, an unexplained recommendation should not be acted upon.

### Hallucination and Verification

Large language models can produce fluent but incorrect output, particularly when used without grounding to verified sources. In allergy, that risk is concrete: a confident but wrong reaction summary will change prescribing. Any deployed system must include retrieval grounding, model auditing, and human verification of any output that affects an allergy entry [39, 42].

### Regulation

The EU AI Act, the EU Medical Device Regulation, the United States Food and Drug Administration’s Software as a Medical Device guidance, and the UK MHRA framework now provide the rules that any clinical computational tool in this space must follow. The relevant requirements, risk classification, traceability, post-market surveillance, and a documented human-oversight role, should be designed into a tool from the start rather than retrofitted before submission.

### Privacy and Generalisability

HIPAA, GDPR, and the equivalent national regimes determine what data can be used for training and how. Federated learning offers a way to train across institutions without moving identifiable data [25]. Even with federated training, NLP models that have to reconcile structured allergy fields with free-text notes need explicit policies for which source is authoritative when they disagree [41]. A reasonable default convention is that the structured field is treated as a hypothesis to be verified, and the free text, once parsed, provides the evidence that supports or refutes it.

### Electronic Health Record Vendors and Interoperability

Successful implementation depends on electronic health record vendors as well as on model developers. Beyond building accurate models, adoption requires that these tools are integrated within existing prescribing workflows and that they interoperate across health systems. The cost of building and maintaining this functionality can be substantial, which creates a barrier to adoption and limits scalability, particularly for resource-constrained institutions. Recognising this shared responsibility among vendors, healthcare organisations, and policymakers is central to any realistic implementation framework.

### Workforce and Training

None of these tools removes the need for clinical staff who can take an allergy history, perform a graded oral challenge, and recognise an immediate hypersensitivity reaction. They do, however, change the shape of the workforce. A pharmacist or a senior nurse using a validated rule-based tool, supported by an NLP-derived history summary and an EHR case-finder, can manage a substantial proportion of the patients who would historically have waited for an allergy clinic slot. Allergy services then concentrate on the moderate and high-risk groups for which specialist judgement is essential. This redistribution is the proximate clinical purpose of the technology and the metric most likely to matter to hospital management.

### Workflow and Culture

A model that produces a correct recommendation that nobody acts on has no clinical effect. The implementation literature in cardiac anaesthesia and intensive care is more mature than in allergy and consistently shows that performance metrics are necessary but not sufficient, clinician trust, alert design, and workflow integration determine whether a tool changes prescribing [27, 28, 43]. The same pattern is now visible in early allergy implementation studies: pathways that pair a computational case-finder with a named clinician responsible for the allergy record outperform those that rely on alerts alone.

## Future Directions

### Multi-centre and Federated Training

The next generation of de-labelling models needs training data from health systems with different documentation styles, languages, and case mixes. Federated learning consortia are the most realistic route, particularly for NLP models that are otherwise tied to one institution’s vocabulary [25, 40].

### Genomic and Immunological Inputs

HLA typing already informs avoidance of specific drugs in specific populations. Combining HLA, transcriptomic, and clinical inputs in a single model is the most promising route for the small but high-stakes group with severe delayed reactions, including DRESS and SJS/TEN [44–46].

### Prospective Trials with Stewardship Endpoints

Most published computational de-labelling work to date has reported model performance, not patient outcomes. The studies that will change practice will measure antibiotic prescribing, infection rates, length of stay, hypersensitivity events, cost, and patient-reported outcomes against a non-computational comparator [25, 41, 46]. A pragmatic stepped-wedge or cluster-randomised design in a single hospital network is now achievable and would generate the kind of evidence that stewardship committees, regulators, and payors expect from any new prescribing intervention. Cost-effectiveness analyses, ideally pre-specified rather than retrofitted, would help position these tools alongside the established economic case for non-allergist de-labelling.

### Low-resource and Remote Applications

Smartphone-based history tools, image triage, and asynchronous specialist review can extend de-labelling to primary care and to settings with no allergist at all [20, 40]. Validation across languages and digital-literacy levels is the precondition.

### Pharmacovigilance

NLP applied to spontaneous reporting and EHR data can flag emerging hypersensitivity signals for new agents, including biologics and vaccines, before population-scale exposure [41, 47]. This is a natural extension of de-labelling work and uses the same NLP infrastructure.

## Conclusion

Penicillin allergy de-labelling is a clinical problem with a known solution that does not scale. Computational methods do not change the diagnostic standard, but they can extend it: they can find the patients who need review, recover the history that was not captured, stratify risk more precisely than fixed rules, suppress unhelpful alerts, and triage cutaneous reactions before a clinic appointment is needed. None of this removes the requirement for clinician judgement, prospective evaluation against stewardship outcomes, and the regulatory and equity safeguards now expected of any clinical computational tool. The current evidence base remains an important caveat: most published studies are single-centre and retrospective and report model performance rather than patient outcomes, and external validation is still limited. With those safeguards in place, the case for embedding these methods into routine penicillin allergy practice, and into the broader drug allergy pathway that follows, is increasingly a question of implementation as much as of remaining evidence gaps. The next generation of work in this field should be judged not by area-under-the-curve on a held-out test set, but by whether more patients receive the right antibiotic and whether fewer carry an inaccurate allergy label out of hospital than would have done without the tool.

## Data Availability

No datasets were generated or analysed during the current study.
